# Supplementary material for: Genetic Diversity and Structure of Rear Edge Populations of Sorbus aucuparia (Rosaceae) in the Hyrcanian Forest
Source: Plants (Basel). 2021 Jul 19;10(7):1471. doi: 10.3390/plants10071471 (PMC8309350; doi:10.3390/plants10071471)
Supplement: Supplementary file 1 [file plants-10-01471-s001.zip › plants-1257446-supplementary.pdf]

Table S1. Repeat motif, primer sequence, fragment size and Tm information for 15 under study microsatellite loci.

Primers name	Name	Sequence (5' ⟶ 3')	Fragment size (bp)
MSS1	F	ATGTTCGGTAGTCATCCCCT	2
	R	GCTCAGATAGCCACTCCCC	2
MSS5	F	CCCCAACAACATTTTTCTCC	2
	R	CCTCTCGCTCTTTGCCTCT	2
MSS6	F	CGAAACTCAAAAACGAAATCAA	2
	R	ACGGGAGAGAAACTCAAGACC	2
MSS9	F	AAGTTTTCAAGCCATTTTCATT	2
	R	CTTCACCATTTTTGTTGTGTGT	2
MSS13	F	TATGCGTCTTTCCATTCCG	2
	R	GCGTTTGACTCACTCAGATTTG	2
MSS16	F	CTCCCCTTGTGTGATGCC	2
	R	TTGCCCTCAAAGAATGCC	2
SA01	F	ATGGAGTTGAGCTCCACATC	2
	R	GGTGGAGGGACAATTGTGTC	2
SA02	F	CTAGGTATCATCTCCGACCA	2
	R	ACGTAGCACTGAATGGTATAG	2
SA03	F	CACTTCTTCCTGCTGTTTGG	2
	R	ACTACTGCTACTTCTGTGGG	2
SA06	F	ATTTGATCCATGTGCGACTGCA	2
	R	TGCAGCGGTTGCAGATTGCA	2
SA07	F	ACGTTTTCAGTATGATGGCC	2
	R	CTTCGCAGTTCATTAAGCAC	2
SA08	F	CAGAGAGAGTGCACTGCCT	2
	R	GAATTCTTGGCAGTTTGCCT	2
SA09	F	CTTGTTGGACGGATTTCTTC	2
	R	CCAATACTTGAGTAGCATAC	2
SA14	F	ATGGATTTAGGTTAACAGTTGTC	2
	R	GAGGTAAAACCTACCAGTATAC	2
SA19.1	F	AAGTTTACAAGAGTGTGTTCAG	2
	R	GAATTCATGAAAGCAGCTAATG	2

Table S2. Null alleles analysis results by Freena.

Locus	Null allele	Global Fst value with ENA correction	Global Fst value without ENA correction
MSS9	0.289	0.008	0.010
MSS16	0.003	0.050	0.050
MSS1	0.043	0.221	0.230
MSS5	0.147	0.250	0.263
MSS13	0.056	0.198	0.203
SA01	0.135	0.052	0.044
SA02	0.057	0.095	0.091
SA08	0.130	0.060	0.065
SA14	0.120	0.011	0.127
Total	0.109	0.121	0.120

Table S3. Migration with correction on the sink population (theta*M)/4

From	BAN	KH	NAV	ASH	LOM
BAN	0	5.536235	6.362446	2.652329	8.067303
KH	3.868974	0	6.064056	5.047637	8.221238
NAV	5.04166	5.957382	0	2.975577	5.084505
ASH	3.243941	4.73483	6.293935	0	13.53416
LOM	3.740837	4.587634	5.806998	3.291832	0
